# Chromatin accessibility analysis suggested vascular induction of the biliary epithelium via the Notch signaling pathway in the human liver

**DOI:** 10.1186/s13104-023-06674-8

**Published:** 2023-12-21

**Authors:** Masaharu Yoshihara, Takahiro Nakayama, Satoru Takahashi

**Affiliations:** 1 Department of Primary Care and Medical Education, Institute of Medicine, University of Tsukuba, Tsukuba, Japan; 2 Laboratory Animal Resource Center, 1-1-1 Tennodai, Tsukuba, Ibaraki 305-8575 Japan; 3 College of Medicine, School of Medicine and Health Sciences, University of Tsukuba, Tsukuba, Japan; 4 Department of Anatomy and Embryology, Institute of Medicine, University of Tsukuba, Tsukuba, Japan; 5 Transborder Medical Research Center, Institute of Medicine, University of Tsukuba, Tsukuba, Japan

**Keywords:** Cholangiocyte, Developmental biology, Epigenetics, Single-cell ATAC-sequencing

## Abstract

The biliary epithelial cells (cholangiocytes) in the liver originate from undifferentiated liver parenchymal cells (hepatoblasts) that are located adjacent to the portal vein. This differentiation process is driven by Notch signaling, which is recognized for generating salt-and-pepper (fine-grained) patterns, in contrast to one- or two-cell layer (spatially confined) patterning in cholangiocyte differentiation. It is unclear how Notch signaling acts and localizes only in cholangiocytes. A computer simulation study suggested that low production rates of the ligands or receptors of Notch signaling are crucial for the spatially confined patterning, although biochemical examination is lacking. Here, we analyzed a publicly available single-cell ATAC-sequencing dataset from human fetal liver samples. We showed high chromatin accessibility for the ligands only in vascular cells, while that for the receptor is limited to a small population of hepatoblasts. This finding strengthens the previously proposed idea that low production rates of the ligands or receptors of Notch signaling enable vascular induction of cholangiocytes.

**Supplementary Information:**

The online version contains supplementary material available at 10.1186/s13104-023-06674-8.

## Introduction

Bile juice is produced by hepatocytes and enters the biliary tree, which is covered with its own epithelial cells named cholangiocytes. Biliary formation in the liver is important, as demonstrated by the end-stage liver disease in a human illness affecting this process (Alagille syndrome) [1]. The components of Notch signaling (JAG1 and NOTCH2) are responsible for this syndrome [2–4]. Indeed, loss-of function mutations or gain-of-function mutations of these components in mice results in decreased or increased biliary formation in the liver [5].

**Fig. 1:**
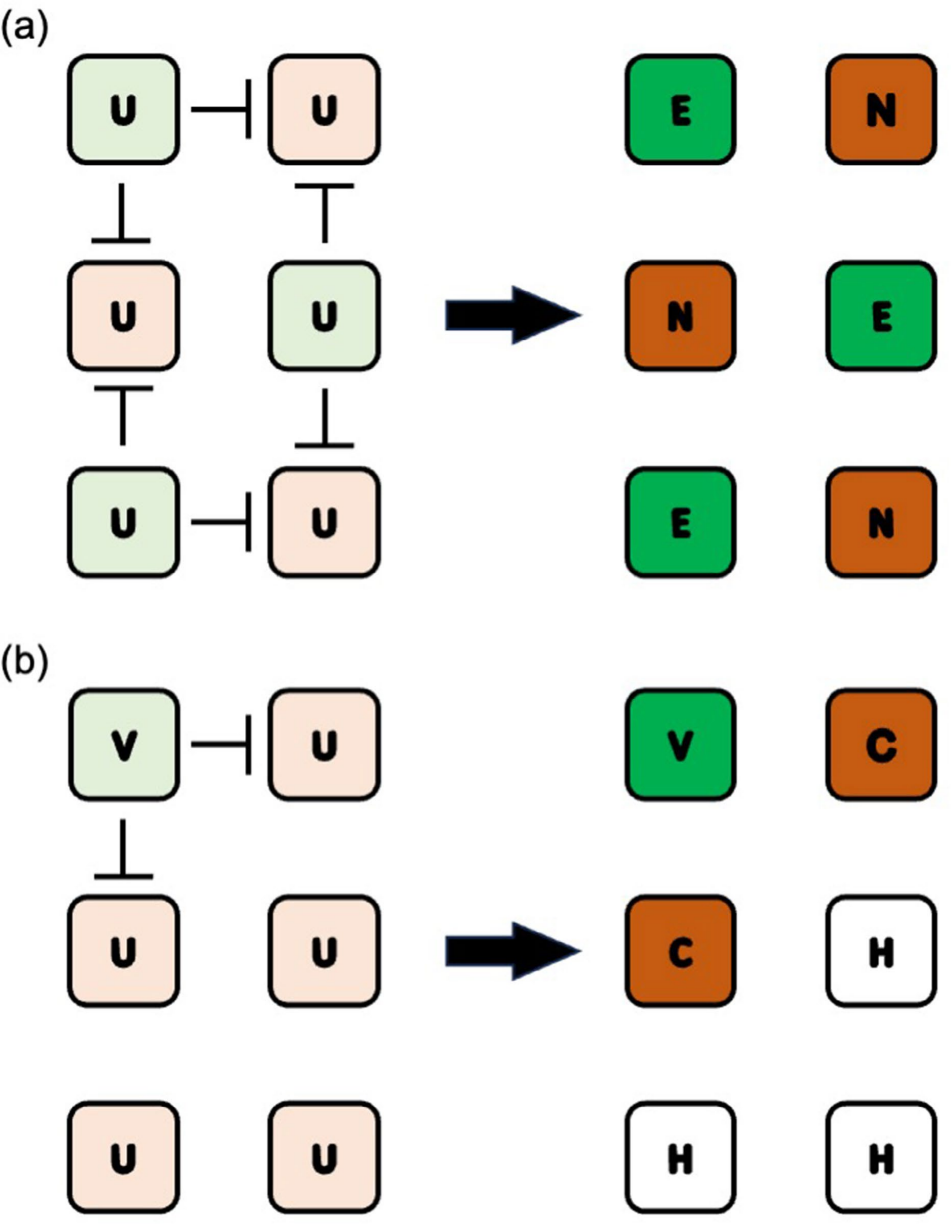
Patterning via the Notch signaling pathway. (**a**) *Drosophila* neurogenesis. The initial state with fluctuation is shown in the left panel (undifferentiated cells; U). Cells slightly colored green express DSL ligands to send Notch signaling and to inhibit the “sender” phenotype in their neighboring cells slightly colored brown. After iteration of cell‒cell communication, sender (green) and receiver (brown) phenotypes become apparent (right panel). The sender cells become epidermal cells (labeled with “E”), whereas the receiver cells become neural cells (labeled with “N”). (**b**) Mammalian biliary development in the liver. Vascular cells (labeled with “V”) transmit Notch signaling to undifferentiated cells (labeled with “U”) in their vicinity. After iteration of cell‒cell communication, the receiver cells become cholangiocytes (labeled with “C”), whereas the others become hepatocytes (labeled with “H”)

Notch signaling is a ligand‒receptor signaling pathway that is evolutionally conserved [6] and, in mammals, is composed of five DSL ligands (JAG1, JAG2, DLL1, DLL3 and DLL4) and four Notch receptors (NOTCH 1, 2, 3, 4) [7]. For example, this signaling is essential for *Drosophila* neurogenesis, which shows a salt-and-pepper (fine-grained) pattern (Fig. 1a) [8]. From this pattern, lateral inhibition with feedback mechanism was proposed, where the production rates of DSL ligands were reduced in Notch signaling-receiving cells. After iterations of cell‒cell communication via the ligands and receptors, the initial fluctuation of the amounts of ligands and receptors among the undifferentiated cells was augmented to show a salt-and-pepper pattern [9]. A computer simulation study suggested that lateral inhibition with feedback mechanism generates the salt-and-pepper pattern when the production rates of the ligands and receptors are high [10]. The same study also suggested that spatially confined patterning via the Notch signaling pathway during biliary formation occurred when either the production rates of the ligands or receptors were low. In this case, the portal veins in the liver act as DSL ligand sources, while Notch signaling is prevented from spreading owing to the low production rates of the ligands or receptors among the undifferentiated cells, resembling the induction phenomenon (Fig. 1b). Indeed, an in vivo examination of DSL ligands and Notch receptors showed vasculature-confined expression of Jag1, supporting this mechanism [11]. In addition, the upstream regulator (Slug) of this vasculature-confined expression of Jag1 was bioinformatically predicted [12]. The biochemical foundation for spatially confined Notch signaling is, however, still unclear. Therefore, in this study, we aimed to examine the cellular capability to express Notch signaling-associated molecules by reanalyzing a publicly available single-cell ATAC-sequencing (scATACseq) dataset from human fetal liver [13].

## Main text

### Methods

#### Animal experiment

CD1(ICR) wild-type mouse fetes at embryonic day (E) 12.5 were collected and fixed in Mildform 10 N (Cat #: 133-10311, FUJIFILM Wako Pure Chemical Corporation, Osaka, Japan). Paraffin-embedded sections (n = 3) were stained with Mayer’s hematoxylin solution (Cat #: 131–09665, FUJIFILM Wako Pure Chemical Corporation) and Eosin Alcohol Solution (Cat #: 050-06041, FUJIFILM Wako Pure Chemical Corporation). Images were captured by using BIOREVO-BZ-X810 (Keyence, Osaka, Japan).

#### Single-cell RNA sequencing of the mouse embryonic livers

The mouse postnatal day 1 liver single-cell RNA sequencing datasets (GSM5239496 and GSM5239497 from GSE171993) [14] were downloaded from the Gene Expression Omnibus database (https://www.ncbi.nlm.nih.gov/geo/). This dataset contained 24,043 features across 9623 cells (n = 2405 and n = 7218) from two mice.

#### ScATACseq of the human fetal livers

The human fetal liver scATACseq dataset (GSM4508935) [13] was downloaded from the Gene Expression Omnibus database (https://www.ncbi.nlm.nih.gov/geo/). In this dataset, an RDS file that contained a processed Seurat object with cell type annotations, for example, was provided. The enriched genes in each cell type were provided in the Gene Expression Omnibus database (GSE149683_ File_S3.motif_enrichment_common_lineages.txt.gz). This dataset contained 1,084,870 features across 183,175 cells from five developmental stages (94 (n = 1), 110 (n = 1), 115 (n = 2), 120 (n = 2) and 122 (n = 1) days of pregnancy) (n = 7 in total). The cell numbers of each developmental stage were 21,653, 35,868, 64,277, 35,447 and 25,930, respectively. For sequencing analysis, we used Seurat version 4.3.0.1 [15] and Signac version 1.10.0 [16] under R version 4.3.1 [17].

## Results

**Fig. 2:**
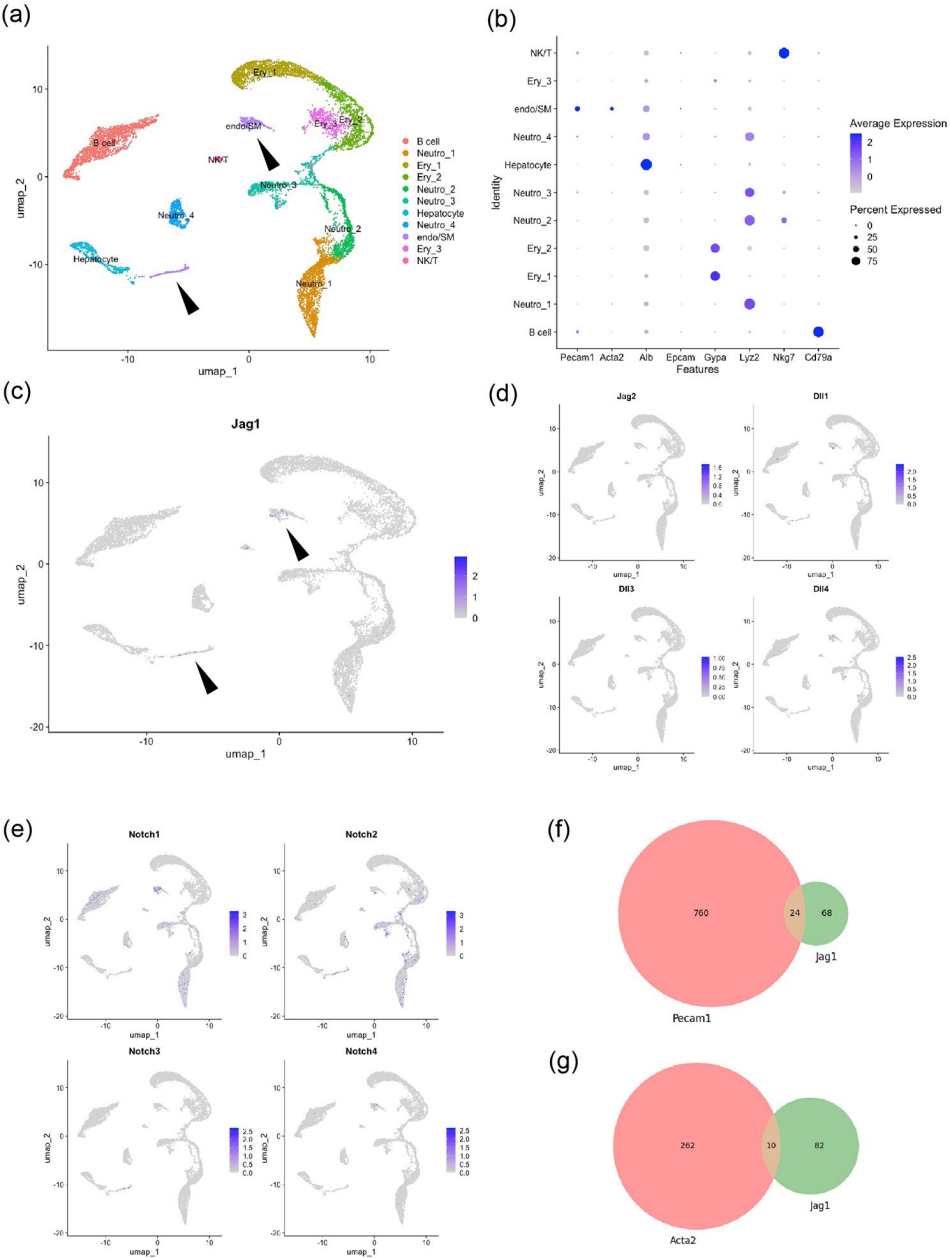
(**a**) Eleven clusters identified by UMAP. Arrowheads: endothelial cell or smooth muscle cell cluster, Neutro: neutrophil, Ery: erythroid, NK/T: NK cells or T cells, endo/SM: endothelial cells or smooth muscle cells. (**b**) Dot plot of marker genes. Note that endo/SM cluster shared Pecam1 (endothelial cell marker) and Acta2 (smooth muscle cell marker) expressions. (**c**) Feature plot of Jag1. Note that Jag1 was expressed in endo/SM cluster (arrowheads). (**d**) Feature plots of Jag2, Dll1, Dll3, and Dll4. (**e**) Feature plots of Notch1, 2, 3, 4. (**f**) Venn diagram of Pecam1- or Jag1- expressing cells. (**g**) Venn diagram of Acta2- or Jag1- expressing cells

To examine expression profiles of the ligands and receptors in Notch signaling pathway, we carried out a reanalysis of a publicly available single-cell RNA sequencing dataset from mouse liver at postnatal day 1 (GSE171993) [14]. Uniform manifold approximation and projection (UMAP) identified eleven clusters including six immune cell clusters, three erythroid clusters, one hepatocyte cluster, and one endothelial/smooth muscle cell cluster (arrowheads) (Fig. 2a and b). The endothelial/smooth muscle cell cluster shared Pecam1 (endothelial cell marker) and Acta2 (smooth muscle cell marker) expressions. Feature plot revealed that Jag1 was expressed in a small population of this endothelial/smooth muscle cell cluster (arrowheads) (Fig. 2c), consistent with previous studies [11, 12]. The other ligands (Jag2, Dll1, Dll3, Dll4) were expressed in a small number of the liver cells (Fig. 2d). At this later developmental stage, Notch1 was expressed in the hepatocyte and neutrophil lineages and Notch2 was mainly expressed in the neutrophil lineage (Fig. 2e). Notch3 and Notch4 were expressed in fewer cells compared with Notch1 and Notch2. We counted the numbers of Pecam1-, Acta2-, Jag1-expressing cells and made Venn diagrams (Fig. 2f, 2 g). Jag1 was expressed in minor populations of both Pecam1-expressing cells and Acta2-expressing cells. Taken together, our reanalysis of single cell RNA sequencing confirmed Jag1 expression in the vascular cells.

**Fig. 3:**
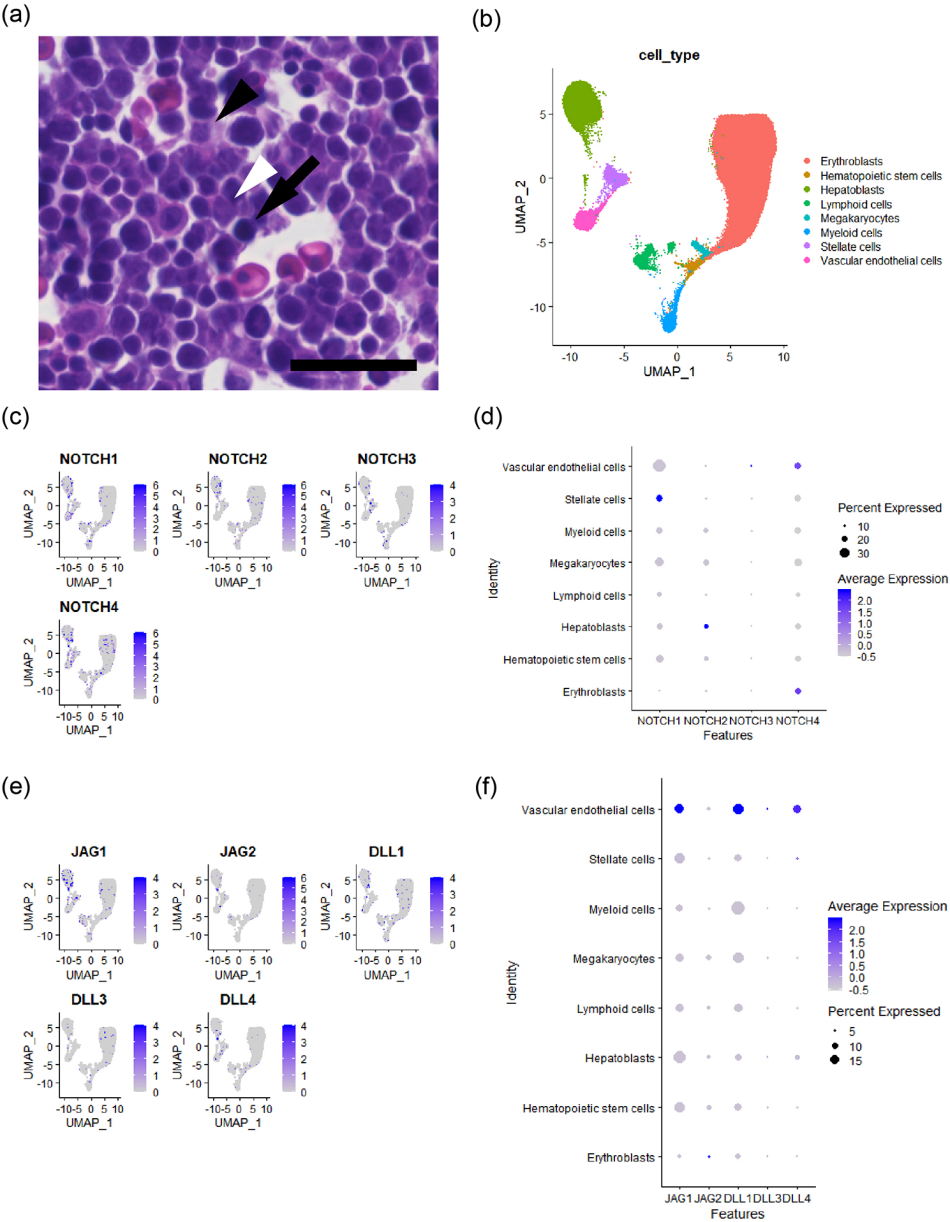
Chromatin landscape of human fetal livers. (**a**) A representative image of the liver of mouse E12.5 embryo. Note that a hepatoblast (white arrowhead) is segregated from a vessel by a hematopoietic cell (arrow) while another hepatoblast (black arrowhead) is neighboring to a vessel. Scale bar = 25 μm. (**b**) UMAP clustering showed large populations of hematopoietic cells, vascular endothelial cells and hepatoblasts. (**c**) Feature plots for chromatin accessibility for the Notch receptors. Note that chromatin accessibility for these genes was generally low except for minor cell populations. (**d**) Dot plot for chromatin accessibility for the Notch receptors. (**e**) Feature plots for chromatin accessibility of the DSL ligands. (**f**) Dot plot for chromatin accessibility of the DSL ligands. Note that the vascular endothelial cells had high chromatin accessibility for JAG1, DLL1 and DLL4

To provide spatial information of the developing liver, we carried out hematoxylin-eosin staining using mouse E12.5 liver (Fig. 3a). The nuclei of hematopoietic cells are condensed compared with those of hepatoblasts [18]. We observed hepatoblasts near (black arrowhead) or far from (white arrowhead) vessels and considerable number of hematopoietic cells (arrow). Importantly, a hepatoblast (white arrowhead) was spatially separated from the vessel by a hematopoietic cell (arrow). UMAP clustering of scATACseq is shown in Fig. 3b. UMAP clustering of each developmental stages is shown in Additional file 1. The liver was mainly composed of hematopoietic cells, hepatoblasts and vascular endothelial cells. Chromatin accessibility for the Notch receptors is shown in feature plots (Fig. 3c) and dot plot (Fig. 3d). We observed small populations of hepatoblasts that had peaks for NOTCH2. Then, chromatin accessibility for the DSL ligands was shown in feature plots (Fig. 3e) and dot plot (Fig. 3f). The vascular endothelial cells had high chromatin accessibility for JAG1, DLL1 and DLL4, which is in contrast to the case with the Notch receptors. Importantly, the majority of hematopoietic cells in this dataset had low levels of chromatin accessibility for DSL ligands and Notch receptors.

## Discussion

The operation mode of Notch signaling has been debated for decades. The early findings from *Drosophila* neurogenesis led to the idea of lateral inhibition with feedback mechanism [9]. In contrast to lateral “inhibition”, lateral “induction”, where Notch signaling triggers the sender phenotype, has been proposed from observations of the developing inner ear [19]. Moreover, *cis*-interactions (interactions within the same cell surface) of the DSL ligands and Notch receptors was reported [20]. Therefore, it would be necessary to identify the operation mode of this signaling pathway when considering a specific biological context.

Biliary development in the liver is unique in that Notch signaling is confined to the hepatoblasts adjacent to the portal vein [10]. Computer simulation predicted low production rates for the DSL ligands or Notch receptors, although the biochemical foundation supporting this idea is lacking. In this study, scATACseq reanalysis revealed that chromatin accessibility for DSL ligands and Notch receptors was generally low except for that for JAG1, DLL1 and DLL4 in vascular endothelial cells. It has not been fully elucidated whether the DSL ligand source is vascular endothelial cells, vascular smooth muscle cells, or their common progenitor, angioblasts, although SM22-Cre-mediated smooth muscle cell-conditional Jag1 knockout resulted in more severe jaundice than VE-Cadherin-Cre-mediated endothelial cell-conditional Jag1 knockout [21]. The findings in the present study, however, are not dependent on the vascular cell types. Biochemical examination, for example, single-cell chromatin immunoprecipitation of the transcription factors responsible for vascular Jag1 expression such as Slug [12], would further test our results. In addition, large scale deletion of open chromatin region of Jag1 in endothelial or smooth muscle cells might be also useful in testing our results.

Two types of stem cell niches have been pointed out in the liver. One is human hepatic stem cells that reside in ductal plates (differentiating biliary epithelium) and in Canals of Hering in fetal and adult livers, respectively [22]. Importantly, survival and proliferation of these cells necessitate paracrine signal from tightly-connecting hepatic stellate cells or angioblasts [22]. Although involvement of Notch signaling in these processes is still unclear, this phenomenon is consistent with the notion that vascular paracrine is essential for the development of biliary tree in the liver. The other one is peribiliary glands where biliary tree stem/progenitors reside near the fibromuscular layer [23], supporting association between undifferentiated cells and smooth muscle cells.

Overall, low chromatin accessibility in hepatoblasts would put constraints on the spread of Notch signaling. In addition, since hematopoietic cells, which are another large population, also have low chromatin accessibility for DSL ligands and Notch receptors, these cells spatially (anatomically) block the spread of Notch signaling. In summary, both epigenetic and anatomical regulation of Notch signaling could be utilized in biliary development in the liver.

## Conclusion

Notch signaling is confined to the hepatoblasts adjacent to the vasculature, partially owing to low chromatin accessibility. Hematopoietic cells might be a positional (anatomical) constraint on the spread of Notch signaling.

### Limitations

First, this study analyzed a scATACseq dataset that lacked positional information. Thus, the effect of positional (anatomical) constraints should be histologically examined. Second, since the samples were collected during mid-pregnancy, the low chromatin accessibility observed here might be a consequence of Notch signaling. Analyzing an earlier sample would resolve this potential problem, although such a dataset is currently unavailable to the best of our knowledge. Third, the analyzed scATACseq dataset is limited to five different timepoints. Excellent (spatial) scATACseq works [24, 25] exist although it was technically difficult for us to carry out reanalysis using these datasets, which would test our results in the present study.

### Electronic supplementary material

Below is the link to the electronic supplementary material.

Supplementary Material 1

## Data Availability

The codes used in the current study are available at our GitHub repository (https://github.com/MasaharuYoshihara/Liver_ATAC).

## Abbreviations

scATACseq: single-cell ATAC sequencing
UMAP: uniform manifold approximation and projection
