# Supplementary material for: Chromatin accessibility analysis suggested vascular induction of the biliary epithelium via the Notch signaling pathway in the human liver
Source: BMC Res Notes. 2023 Dec 21;16:379. doi: 10.1186/s13104-023-06674-8 (PMC10734141; doi:10.1186/s13104-023-06674-8)
Supplement: Supplementary file 1 — Supplementary Material 1 [file 13104_2023_6674_MOESM1_ESM.pdf]

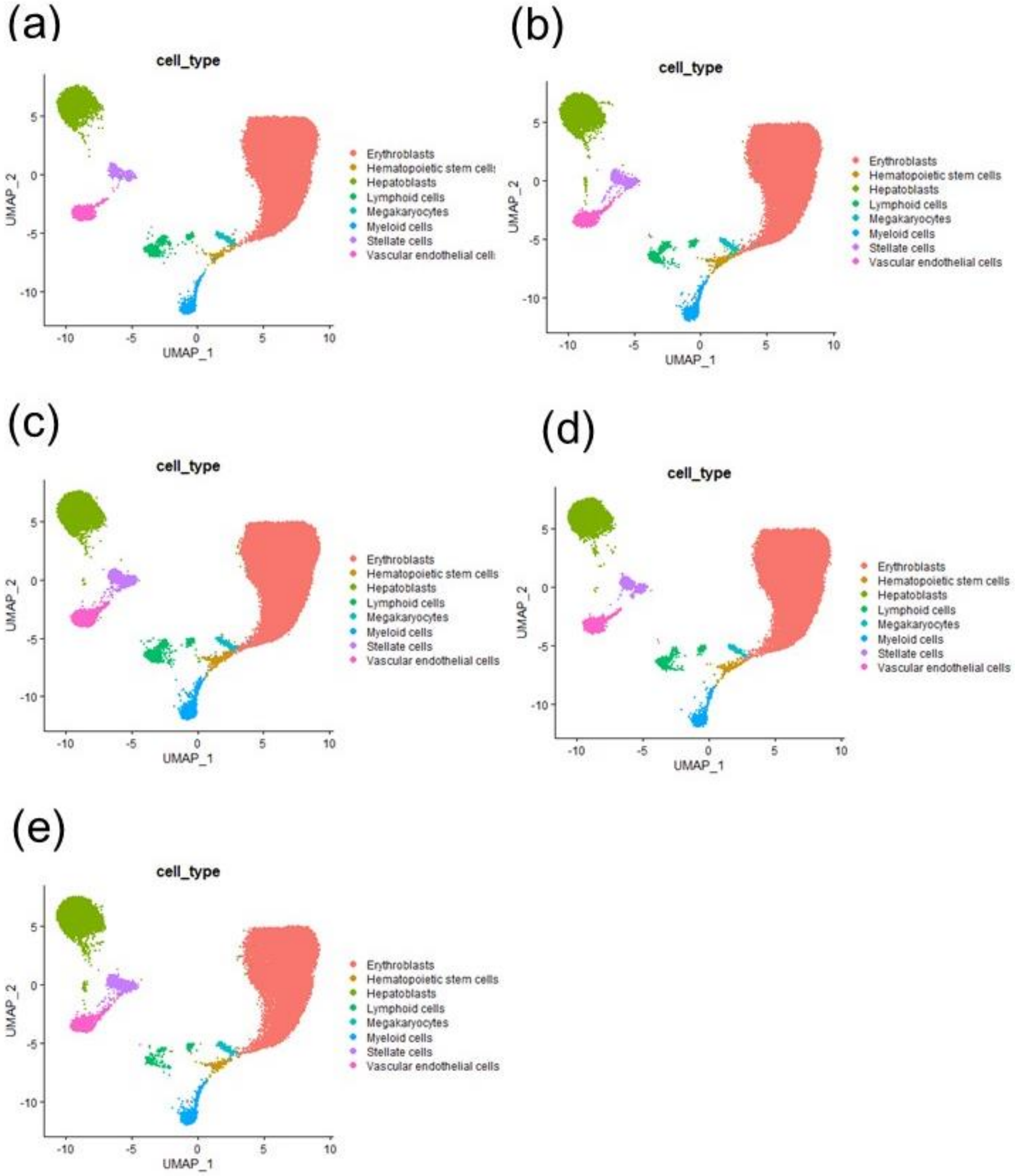

Supplementary file

File name: Additional_file_1

File format: .pdf

Title of data: UMAP clustering of the five developmental stages

Description of data: (a) 94 days of pregnancy. (b) 110 days of pregnancy. (c) 115 days of pregnancy. (d) 120 days of pregnancy. (e) 122 days of pregnancy. Note that all five samples showed similar UMAP clustering results.
